# Female mice lacking cholecystokinin 1 receptors have compromised neurogenesis, and fewer dopaminergic cells in the olfactory bulb

**DOI:** 10.3389/fncel.2013.00013

**Published:** 2013-03-01

**Authors:** Yi Sui, Rob Vermeulen, Tomas Hökfelt, Malcolm K. Horne, Davor Stanić

**Affiliations:** ^1^Neurodegeneration Division, Florey Institute of Neuroscience and Mental Health, University of MelbourneParkville, VIC, Australia; ^2^Department of Neurology, Shenyang First People's HospitalShenyang, China; ^3^Department of Neuroscience, Karolinska InstitutetStockholm, Sweden; ^4^Department of Neuroscience, Universiteit MaastrichtMaastricht, Netherlands; ^5^Neurology Department, St Vincent's HospitalFitzroy, VIC, Australia; ^6^Department of Florey Neuroscience, University of MelbourneMelbourne, VIC, Australia

**Keywords:** cholecystokinin 1 receptor, neurogenesis, subventricular zone, rostral migratory stream, olfactory bulb, subgranular zone, interneurons, survival

## Abstract

Neurogenesis in the adult rodent brain is largely restricted to the subependymal zone (SVZ) of the lateral ventricle and subgranular zone (SGZ) of the dentate gyrus (DG). We examined whether cholecystokinin (CCK) through actions mediated by CCK1 receptors (CCK1R) is involved in regulating neurogenesis. Proliferating cells in the SVZ, measured by 5-bromo-2-deoxyuridine (BrdU) injected 2 h prior to death or by immunoreactivity against Ki67, were reduced by 37 and 42%, respectively, in female (but not male) mice lacking CCK1Rs (CCK1R^−/−^) compared to wild-type (WT). Generation of neuroblasts in the SVZ and rostral migratory stream (RMS) was also affected, since the number of doublecortin (DCX)-immunoreactive (ir) neuroblasts in these regions decreased by 29%. In the SGZ of female CCK1R^−/−^ mice, BrdU-positive (+), and Ki67-ir cells were reduced by 38 and 56%, respectively, while DCX-ir neuroblasts were down 80%. Subsequently, the effect of reduced SVZ/SGZ proliferation on the generation and survival of mature adult-born cells in female CCK1R^−/−^ mice was examined. In the OB granule cell layer (GCL), the number of neuronal nuclei (NeuN)-ir and calretinin-ir cells was stable compared to WT, and 42 days after BrdU injections, the number of BrdU+ cells co-expressing GABA- or NeuN-like immunoreactivity (LI) was similar. Compared to WT, the granule cell layer of the DG in female CCK1R^−/−^ mice had a similar number of calbindin-ir cells and BrdU+ cells co-expressing calbindin-LI 42 days after BrdU injections. However, the OB glomerular layer (GL) of CCK1R^−/−^ female mice had 11% fewer NeuN-ir cells, 23% less TH-ir cells, and a 38% and 29% reduction in BrdU+ cells that co-expressed TH-LI or GABA-LI, respectively. We conclude that CCK, via CCK1Rs, is involved in regulating the generation of proliferating cells and neuroblasts in the adult female mouse brain, and mechanisms are in place to maintain steady neuronal populations in the OB and DG when the rate of proliferation is altered.

## Introduction

Adult olfactory precursors divide primarily within the subventricular or subependymal zone (SVZ) of the lateral ventricle, where they differentiate into immature neurons. Neuroblasts then migrate tangentially along the rostral migratory stream (RMS) toward the main olfactory bulb (OB) (Curtis et al., 2007). When neuroblasts reach the OB, they migrate radially into the granular (GCL) and periglomerular (GL) layers of the OB, where they differentiate into local interneurons (Luskin, 1993; Lois and Alvarez-Buylla, 1994). In the dentate gyrus (DG), neural progenitors in the subgranular zone (SGZ) proliferate and give rise to immature neurons (Altman and Das, 1965; Eriksson et al., 1998; Van Praag et al., 2002) that migrate a short distance to the granule cell layer (GrDG), where they functionally integrate into hippocampal circuitry (Kempermann et al., 2003).

In the young adult rodent, approximately 50% of adult-born cells that migrate into the OB differentiate to form interneurons that integrate into OB circuitry, while the other half undergo programmed cell death as progenitors, neuroblasts or young neuronal cells in the SVZ, RMS, or OB (Petreanu and Alvarez-Buylla, 2002; Winner et al., 2002; Lledo and Saghatelyan, 2005). Doublecortin (DCX), a neuron specific microtubule associated protein, is expressed by most dividing neuroblast cells and migrating postmitotic neuroblasts in the SVZ and RMS, and its downregulation 10–14 days after the birth of a neuroblast coincides with the commencement of neuronal nuclei (NeuN) expression, as the cells mature to become OB interneurons (Brown et al., 2003). Similarly, 50% of newly generated cells in the adult rodent GrDG die within 22 days of their birth (Dayer et al., 2003). DCX is expressed by adult-born cells on days 1–14 after birth (Brown et al., 2003), with some cells being NeuN-positive (+) on day 1 (Brandt et al., 2003), and the majority of surviving cells expressing NeuN (Brown et al., 2003) and calbindin (Brandt et al., 2003) 1 month after birth.

A wide range of molecular cues regulate neurogenesis in the adult brain (Lie et al., 2004; Abrous et al., 2005; Emsley et al., 2005; Ming and Song, 2005), and peptidergic systems, including neuropeptide Y (Hansel et al., 2001; Howell et al., 2005; Hökfelt et al., 2008; Stanić et al., 2008) and galanin (Mazarati et al., 2004), also participate in these processes. A neuropeptide that has remained largely unexplored within this context, and which may potentially regulate neurogenesis, is cholecystokinin (CCK). CCK is widely distributed in the mammalian CNS (Vanderhaeghen et al., 1975; Hökfelt et al., 1988) and so far two distinct CCK receptors have been cloned; the CCK 1 (CCK1R) and CCK 2 receptor (Hill et al., 1987; Wank et al., 1992). CCK modifies the migratory abilities, proliferation, and survival of tumor astrocytes (De Hauwer et al., 1998; Lefranc et al., 2002) and lymphocytes (Medina et al., 1998), and guides migrating gonadotropin-releasing hormone-1 (GnRH-1) neuroendocrine neurons into the brain (Giacobini et al., 2004). Moreover, immortalized rat brain neuroblasts express CCK1R and CCK2R mRNA (Langmesser et al., 2007). Exposure to CCK promoted proliferation of these cells, and improved their viability, indicating that CCK is an important regulator of proliferation.

We therefore examined whether CCK is involved in regulating neurogenesis in the adult brain. Using adult mice with genetic deletion of the CCK1 receptor (CCK1R^−/−^), we investigated whether CCK1 receptors influence cell proliferation and neuroblast formation in the SVZ, RMS, and SGZ, and affect interneuron generation in the OB and DG. We report that female, but not male, CCK1R^−/−^ mice have fewer proliferating cells, migratory neuroblasts, and tyrosine hydroxylase (TH)-immunoreactive (ir) OB interneurons than wild-type (WT) mice.

## Materials and methods

### Animals

All experimental procedures conformed to the Australian National Health and Medical Research Council published code of practice, and were approved by the Florey Neuroscience Institutes' Animal Ethics Committee (#09-053 and #07-117). Twelve female and four male 16–20-week old mice lacking the CCK1R (Strain Name: 129-*Cckar*^*tm1Kpn*^/J; Stock No. 006367; The Jackson Laboratory, Bar Harbor, ME) (Kopin et al., 1999) and 12 female and 4 male age-matched WT control mice, weighing between 20–25 g were used. All animals were maintained under standard conditions on a 12 h day/night cycle, with water and food *ad libitum*.

### Genotyping

To obtain genomic DNA, 2–5 mm mouse tails were digested in 100 μL proteinase K solution (250 μL Tween 20, 500 μL 1M Tris, 2500 μL × 20 mg/ml proteinase K and MQ water to 50 ml). Mixtures were then incubated at 56°C for 60–90 min, followed by 10 min at 95°C. Tubes were centrifuged at maximum speed for 10 min, and subsequently stored at 4°C. For each PCR, 1 μl DNA template was added to 6 μl 2×Go Taq Green polymerase master mix (Promega, Madison, WI, Code No. 9PIM712), 1 μl of 10 μM mixture of each primer and 4 μl nuclease free water (Promega, Code No. P1193). PCR was performed on a T3 Thermocycler (Biometra, Göttingen, Germany) with the following primer sequences (Geneworks, Hindmarsh, Australia): 5′-GCT GCA TAG CGT CAC TTG G-3′ for CCK1 receptor WT forward; 5′-GAT GGA GTT AGA CTG CAA CC-3′ for CCK1 receptor WT reverse; 5′-GAC AAT CGG CTG CTC TGA TG-3′ for CCK1R^−/−^ forward. Cycling conditions were: 95°C for 5 min for initial denaturation, 35 cycles of 95°C for 30 s, 63°C for 60 s, and 72°C for 60 s, followed by final amplification at 72°C for 5 min. Final DNA products were visualized under UV after electrophoresis in 1.5% agarose gel containing 0.5 μL SYBR Safe DNA gel stain (Invitrogen, Carlsbad, CA, Code No. S33102) per 10 ml. The expected fragments yielded by PCR were 507 bp for WT, 970 + 507 bp for CCK1R ± and 970 bp for CCK1R^−/−^.

### BrdU administration

**Figure 1:**
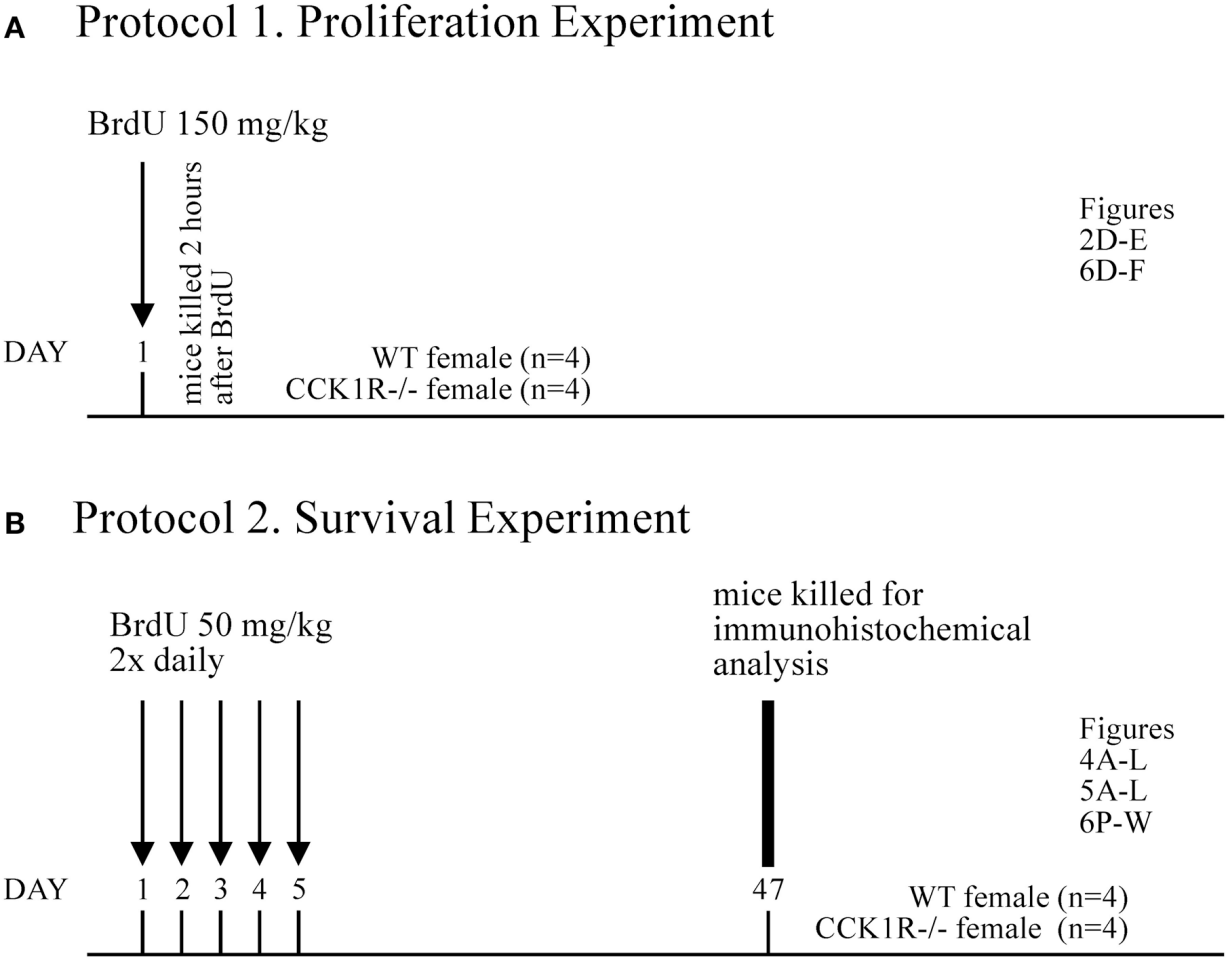
**BrdU Experimental Design. (A)** *Protocol 1*. To identify proliferating cells in the SVZ and SGZ, a single dose of BrdU (150 mg/kg i.p.) was administered 2 h prior to death (*n* = 4 for each experimental group). From these animals, the number of BrdU+ cell bodies in the SVZ (Figures 2D,E) and SGZ (Figures 6A–C) was estimated. **(B)** *Protocol 2*. To label mature adult-born cells that migrate to, integrate and survive in the GCL and GL of the OB, and the GrDG of the hippocampal formation, BrdU (50 mg/kg, i.p.) was administered twice daily for 5 consecutive days, and mice killed 42 days later (i.e., 47 days after first BrdU administration; *n* = 4 for each experimental group). From these animals, the number of BrdU+ cell bodies in the GCL (Figures 4A,B) and GL (Figures 5A,B) of the OB was estimated, as was the number of: BrdU/GABA (Figures 4C–E), BrdU/NeuN (Figures 4G–I), and BrDU/calretinin (Figures 4J–L) co-expressing cells in the GCL of the OB; BrdU/TH (Figures 5C–F), BrdU/GABA (Figures 5G–I), and BrdU/calbindin (Figures 5J–L) co-expressing cells in the GL; BrdU+ cells in the GrDG (Figures 6P–R); and BrdU/calbindin co-expressing cells in the GrDG (Figures 6S–W).

5-bromo-2-deoxyuridine (BrdU) (ICN Biomedicals Inc, Aurora, OH, Cat No. 100171) was administered intraperitoneally to CCK1R^−/−^ and WT mice to study the proliferation and survival of adult-born cells in the SVZ, OB and DG of the hippocampal formation. Two different protocols were used to identify either proliferating cells in the SVZ and SGZ, or “mature” cells that had survived or integrated into the OB or GrDG: (1) To enable identification of proliferating cells in the SVZ/SGZ, a single dose of BrdU (150 mg/kg, i.p.) was injected 2 h prior to their death (Figure 1A); and (2) To label mature adult-born cells that migrate to, integrate and survive in the GCL, GL or GrDG, BrdU (50 mg/kg, i.p.) was administered twice daily for 5 consecutive days, and mice killed 42 days later (Figure 1B).

### Tissue preparation

All animals were deeply anaesthetized using pentobarbitone sodium (Lethabarb, Virbac, Milperra, NSW, Australia, 100 mg/kg i.p.) and perfused through the heart via the ascending aorta with 20 ml Ca^2+^-free Tyrode's buffer (37°C), followed by 20 ml of a mixture of 4% paraformaldehyde (Sigma-Aldrich, St. Louis, MO) and 0.2% picric acid (Sigma) diluted in 0.16 M phosphate buffer (pH 6.9, 37°C) (Pease, 1962; Zamboni and De Martino, 1967) and 50 ml of the same fixative at 4°C, the latter for approximately 5 min. The brains were dissected out and postfixed in the same fixative for 90 min at 4°C, and finally immersed for 48 h at 4°C in 10% sucrose dissolved in phosphate buffered saline (PBS, pH 7.4) containing 0.01% sodium azide (Sigma) and 0.02% bacitracin (Sigma), before rapid freezing by CO_2_. Sections were cut using a cryostat (Leica CM1850, Wetzlar, Germany) at: (1) a thickness of 14 microns, and thaw-mounted on slides coated with 0.5% gelatin (Sigma) and 0.05% chromium(III) potassium sulphate dodecahydrate (Merck, KGaA, Darmstadt, Germany); or (2) a thickness of 30 microns, and stored in a cyroprotectant solution [30% v/v ethyleneglycol (Merck); 15% w/v sucrose; 35% v/v 0.1 M phosphate buffer; 35% v/v distilled H_2_O], at −20°C.

### Immunohistochemistry

#### Incubation protocol (immunofluorescence)

Sections were washed using 0.01 M PBS (3 × 10 min) and incubated for 24 h at 4°C with a rat anti-BrdU (1:300, Axyll, Westbury, NY, Code No. OBT0030), rabbit anti-calbindin (1:10,000, Swant, Marly, Switzerland, Code No. CB-38a), goat anti-calretinin (1:4000, Swant, Code No. CG1), goat anti-DCX (1:1000, Santa Cruz Biotechnology, Santa Cruz, CA, Code No. SC-8066), rabbit anti-γ-aminobutyric acid (GABA) (1:2000, Sigma, Code No. A2052), rabbit anti-glial fibrillary acidic protein (GFAP) (1:400, Dako, Glostrup, Denmark, Code No. Z0334), mouse anti-NeuN (1:1000, Millipore, Billerica, MA, Code No. MAB377), rabbit anti-tyrosine hydroxylase (TH) (1:1000, Pel-Freeze, Rogers, Ar, Code No. P40101-0) or sheep anti-TH (1:400, Pel-Freeze, Code No. P60101-0) antibody, diluted in 0.01 M PBS containing 0.3% Triton X-100 and 0.5% BSA. Sections were then washed in TNT buffer [0.1 M Tris-HCl, pH 7.5; 0.15 M NaCl; 0.05% Tween 20 (Sigma)] for 15 min and incubated in TNB buffer [0.1 M Tris-HCl, pH 7.5; 0.15 M NaCl; 0.5% blocking reagent (PerkinElmer, Boston, MA, Code No. FP1020)] for 30 min at room temperature (RT). Immunoreactivity was visualized using Alexa Fluor 594-conjugated goat anti-rat, Alexa Fluor 488-conjugated donkey anti-goat, Alexa Fluor 594-conjugated goat anti-rabbit, Alexa Fluor 488-conjugated goat anti-rabbit, Alexa Fluor 594-conjugated goat anti-mouse or Alexa Fluor 488-conjugated donkey anti-sheep (1:200, Molecular Probes, Eugene, OR), as appropriate, in TNB buffer for 2 h. Finally, sections were washed in TNT (3 × 10 min) and coverslipped using a fluorescent mounting medium (Dako). Hoechst 33342 (1:1000, Invitrogen) was applied to sections immunostained with GFAP for 1.5 min during the third TNT wash, to provide a nuclear counter stain. Prior to commencing immunoreactivity for BrdU, antigen retrieval and DNA denaturation was performed, where sections were incubated in 50% formamide (BDH Laboratory Supplies, England) in 0.01 M PBS at 65°C for 2 h, 2 M HCl for 30 min at 37°C, and 0.1 M sodium borate (Borax, Sigma, B-3545) buffer for 10 min at RT.

To visualize calbindin and calretinin immunoreactivity, sections were processed using a commercial kit (TSA^+^, NEN Life Science Products, Inc., Boston, MA). Briefly, following 24 h incubation in primary antisera, sections were washed in TNT buffer (15 min), incubated with TNB buffer (30 min) and incubated with horse-radish peroxidase (HRP)-conjugated swine anti-rabbit (1:200, Dako) or HRP-conjugated donkey anti-goat (1:500, Jackson ImmunoResearch Laboratories, West Grove, PA), as appropriate, diluted in TNB buffer for 30 min. Sections were then washed in TNT buffer (3 × 10 min) and incubated in a biotinyl tyramide-fluoroscein (BT-FITC) conjugate (NEN) diluted 1:100 in amplification diluent for 10 min at RT, followed by washes in TNT (3 × 10 min).

For double-immunofluorescence experiments, antigen retrieval and DNA denaturation was performed first, followed by incubations of the anti-BrdU and either the anti-calbindin, calretinin, DCX, GABA, NeuN, or TH antibodies, according to the above concentrations and protocols.

#### Incubation protocol [diaminobenzidine (DAB)]

Sections were rinsed (3 × 10 min) in 0.01 M PBS, followed by incubation in blocking diluent [0.01 M PBS containing 5% normal goat serum (NGS) and 0.3% Triton X-100 (Sigma)] for 30 min, and rabbit anti-Ki67 antibody (1:15,000, Thermo Fisher Scientific, Fremont, CA, Code No. RM-9106-s1) diluted in 0.01 M PBS, 1% NGS and 0.3% Triton X-100 for 48 h at 4°C. Sections were then incubated in biotinylated goat anti-rabbit (1:1000, Dako) diluted in 0.01 M PBS, 1% NGS and 0.3% Triton X-100 for 3 h at RT, and then avidin peroxidase (1:5000 in 0.01 M PBS and 0.075% Triton X-100) for 1 h, followed by DAB (1:100, Sigma) for 20 min. Three percent Hydrogen peroxidase (Merck) was added to the DAB solution for substrate precipitation and the reaction terminated 2 min later by rinsing sections in 0.01 M PBS. Sections were counter stained with neutral red, dehydrated in a series of graded ethanol (50–100%), cleared in X3B solvent (Shell Chemicals, Hawthorn East, Australia), and then coverslipped with DePeX (VWR International, Poole, England). Rinses using 0.01 M PBS (3 × 10 min) were performed between each step.

### Image processing

After processing, sections were examined using a Leica DMLB2 fluorescence microscope (Leica, Wetzlar, Germany), equipped with a dark field condenser and epi-polarization, and epifluorescence with appropriate filter combinations, and with objective lenses of ×10 (N.A. 0.45), ×20 (N.A. 0.70), ×40 (N.A. 0.75), ×60 oil (N.A. 1.40), and ×100 oil (N.A. 1.30). Photographs were taken using a Microfire digital camera (2.3A, Optronics, Goleta, CA) attached to the microscope, operated through Picture Frame software (v2.3, Optronics). For confocal analysis, an Olympus FV1000 confocal laser scanning microscope equipped with ×10 (N.A. 0.4), ×20 (N.A. 0.75), ×40 oil (N.A. 1.30) and ×60 oil (N.A. 1.35) objectives was used. The AlexaFluor 488 and FITC labeling was excited using the 473 nm diode laser. For the detection of AlexaFluor 594, a 559 nm diode laser was used. Z-stack images were captured with multiple images, each separated by a stepwise depth of 1.0 um in the z-plane. Digital images from the microscopy were slightly modified to optimize for image resolution, brightness and contrast using Adobe Photoshop CS5 software (Adobe Systems Inc., San Jose, CA), so as to best represent the immunohistochemistry observed at the microscope.

### Stereology

For quantification of cell bodies in the SVZ and RMS, the level at which the anterior commissure (AC) converged through the midline [Bregma +0.14 mm (see Paxinos and Franklin, 2001)] was used as a reference to define the caudal boundary of the SVZ. Serial sections rostral to this point were acquired, with sections between 0–1400 μm rostral to the AC convergence (i.e., Bregma +0.14 to +1.54 mm) defined as containing the SVZ, and sections from 1500 to 4100 μm (i.e., Bregma +1.6 to +4.2 mm) regarded as having the RMS. Analysis of the OB was performed on sections rostral to Bregma +2.6 mm. Fourteen μm-thick sections, each 280 μm apart, were analysed, and guard zones of 1 μm (top) and 1 μm (bottom) were employed. For quantification of proliferating cells and neuroblasts in SVZ, only the lateral wall of the lateral ventricle was analyzed, as these cells are largely absent in the medial and dorsal walls (Doetsch et al., 1997).

Regions of the DG from which cell bodies were quantified corresponded to Bregma −1.30 to −3.10 mm (see Paxinos and Franklin, 2001). DCX-ir and Ki67-ir cell bodies in the SGZ/GrDG were counted on 14 μm-thick sections, each 140 μm apart, and GFAP-ir and calbindin-ir cell bodies on 14 μm-thick sections, each 280 μm apart. BrdU+ in the SGZ/GrDG, and BrdU+ cell bodies expressing calbindin-like immunoreactivity (LI), were quantified on 30 μm-thick free floating sections, each 180 μm apart, with guard zones of 1 μm (top and bottom). For quantification of Ki67-ir and BrdU-positive proliferating cells in the SGZ, the SGZ was recognized as the border between the polymorph and granule cell layers of the DG, including one cell body width of the GrDG and the equivalent of two granule cell body widths within the polymorph layer (Cameron and McKay, 2001).

Estimates of the number of proliferating cells, neuroblasts and mature neurons in the SVZ, RMS, OB, and DG were made using a fractionator sampling design according to optical dissector rules (Gundersen et al., 1988; West et al., 1991; Stanic et al., 2003; Parish et al., 2005). Regular predetermined *x*, *y* intervals and counting frame dimensions for all estimates were derived by means of a grid program (Stereoinvestigator v.7.0, MicroBrightField, Williston, VT, viewed through a microscope, Leica) and are outlined in Table 1.

**Table 1:** **Counting frame dimensions and *x*, *y* co-ordinates for estimates of proliferating cells (Ki67, BrdU 2 h), migrating neuroblasts (DCX), interneurons, and mature cells (NeuN, TH, calbindin, calretinin, GABA, BrdU 42 days) in the SVZ, RMS, OB, SGZ, and GrDG**.

**Antibody**	**Region analysed**	**Counting frame size (μm)**	**Fractionator *x*, *y* coordinates (μm)**
BrdU (2 h)	SVZ	30 × 30	30 × 30
Ki67	SVZ/RMS	30 × 20	40 × 100
DCX	SVZ/RMS	30 × 20	70 × 150
GFAP	SVZ	40 × 40	40 × 40
NeuN	GCL	20 × 20	70 × 300
NeuN	GL	30 × 30	70 × 300
Calretinin	GCL	80 × 80	150 × 300
Calbindin, Calretinin	GL	80 × 80	100 × 400
GABA, TH, GABA/TH	GL	40 × 40	100 × 400
BrdU (42 days)	GCL	50 × 50	100 × 200
BrdU (42 days)/GABA	GCL	50 × 50	100 × 200
BrdU (42 days)/NeuN	GCL	50 × 50	100 × 200
BrdU (42 days)/Calretinin	GCL	50 × 50	100 × 200
BrdU (42 days)/DCX	GCL	50 × 50	100 × 200
BrdU (42 days)	GL	170 × 135	170 × 270
BrdU (42 days)/TH	GL	170 × 135	170 × 270
BrdU (42 days)/GABA	GL	170 × 135	170 × 270
BrdU (42 days)/Calbindin	GL	170 × 135	170 × 270
BrdU (42 days)/DCX	GL	170 × 135	170 × 270
BrdU (2 h)	SGZ	170 × 135	170 × 135
BrdU (42 days)	GrDG	170 × 135	170 × 135
BrdU (42 days)/Calbindin	GrDG	170 × 135	170 × 135

### Statistical analysis

Data were analyzed using GraphPad Prism 4 (GraphPad Software, San Diego, CA). All comparisons were conducted by student *t*-tests, and ANOVA with Tukey multiple comparisons test where indicated, and a value of *p* < 0.05 was considered statistically significant. Values are expressed as the mean ± SEM.

## Results

We examined whether the number of proliferating cells in the SVZ and SGZ was altered in adult mice lacking the CCK1R. Subsequently, stereological quantification was performed to estimate: (1) the number of migrating neuroblasts in the SVZ and RMS; (2) the number of mature interneurons present in the OB; and (3) the survival of adult-born cells in the OB. Also, we examined whether the number of proliferating cells, neuroblasts and mature neurons was altered in the DG of CCK1R^−/−^ mice.

### Adult female CCK1R^−/−^ mice have a lower number of proliferating cells in the SVZ and RMS

**Figure 2:**
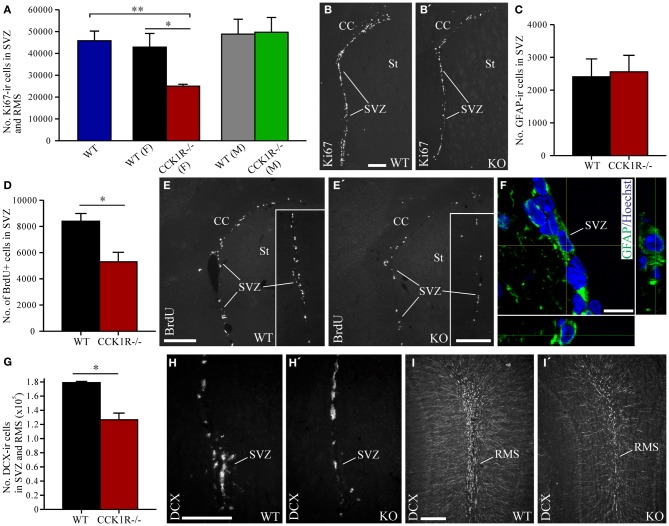
**Estimates of proliferating cells, neuroblasts, and astrocytes in the SVZ and RMS of CCK1R^−/−^ and wild-type mice. (A,B)** Estimated number of Ki67-ir cell bodies in the SVZ and RMS of CCK1R^−/−^ and wild-type mice **(A)**. In **(A)** WT blue bar = wild-type (male and female combined, *n* = 8); WT (F) black bar = female wild-type (*n* = 4); CCK1R^−/−^ (F) red bar = female CCK1R^−/−^ (*n* = 4); WT (M) grey bar = male wild-type (*n* = 4); and CCK1R^−/−^ (M) green bar = male CCK1R^−/−^ (*n* = 4). Photomicrographs of Ki67-LI in the SVZ of female **(B)** wild-type and **(B′)** CCK1R^−/−^ mice. **(D,E)** Estimated number of BrdU+ cell bodies in the SVZ of female CCK1R^−/−^ and wild-type mice **(D)** (see *Protocol 1*, Figure 1A). Photomicrographs of BrdU-LI in the SVZ of female **(E)** wild-type and **(E′)** CCK1R^−/−^ mice. **(C,F)** Estimated number of GFAP-ir astrocytes in the SVZ of female wild-type and CCK1R^−/−^ mice **(C)**. Double-immunofluorescence confocal micrograph of GFAP-LI (green) and Hoechst staining (blue) in the SVZ of female CCK1R^−/−^ mouse **(F)**, the latter providing a nuclear counter stain. **(G–I)** Estimated number of DCX-ir neuroblasts in the SVZ and RMS of female wild-type and CCK1R^−/−^ mice **(G)**. Photomicrographs of DCX-ir neuroblasts in the **(H,H′)** SVZ and **(I,I′)** RMS of female wild-type and CCK1R^−/−^ mice, respectively. In plots **(A,C,D,G)**, black bars = female wild-type animals (*n* = 4), and red bars = female CCK1R^−/−^ mice (*n* = 4). CC, corpus collosum; ir, immunoreactive; KO, CCK1R^−/−^; RMS, rostral migratory stream; St, striatum; SVZ, subventricular zone; WT, wild-type; +, postive. Scale bars: **(B)** = 200 μm, applies **(B,B′)**; **(E)** = 200 μm, applies **(E,E′)**; **(E′)** inset = 200 μm, applies **(E)** inset, **(E′)** inset; **(F)** = 10 μm; **(H)** = 200 μm, applies **(H,H′)**; **(I)** = 200 μm, applies **(I,I′)**. ^*^corresponds to *P* < 0.05 (student *t*-test); ^**^corresponds to *P* < 0.05 (ANOVA with Tukey multiple comparisons test).

Immunoreactivity against Ki67, which labels cells in all phases of mitosis, except G1, was used to identify dividing cells in the SVZ and RMS. There were 42% fewer Ki67-ir cell bodies in female CCK1R^−/−^ mice than in female WT mice (Figures 2A,B). In contrast, the number of Ki67-ir proliferating cells was similar in male CCK1R^−/−^ and male WT mice (Figure 2A). ANOVA with Tukey multiple comparisons test indicated a statistical difference between WT and female CCK1R^−/−^ mice and female and male CCK1R^−/−^ mice, and a statistical similarity between WT mice and male CCK1R^−/−^ mice, and male and female WT mice (Figure 2A).

The rate of cell proliferation in the SVZ was further examined by injecting BrdU (150 mg/kg i.p.) into mice 2 h prior to their death, to label cells in S-phase of the cell cycle (Figure 1A). The number of BrdU-positive (BrdU+) cell bodies in the SVZ of female CCK1R^−/−^ mice was 37% lower than the number in female WT mice (Figures 2D,E).

### Lower numbers of DCX-ir neuroblasts in the SVZ and RMS of female CCK1R^−/−^ mice

Because cell proliferation in the SVZ and RMS of female CCK1R^−/−^ mice was reduced, we next examined whether the number of neuroblasts in the SVZ and RMS were altered. Neuroblasts in these regions were identified by immunoreactivity against DCX (Francis et al., 1999; Gleeson et al., 1999; Brown et al., 2003). The number of DCX-ir neuroblasts in the SVZ and RMS decreased by 29% in CCK1R^−/−^ mice (Figures 2G–I).

### No change in the number of GFAP-ir astrocytes in the SVZ of female CCK1R^−/−^ mice

Immunohistochemistry for GFAP was performed to determine whether reduced cell proliferation in the SVZ had an effect on the number of astrocytes (Merkle et al., 2004). The number of GFAP-ir cell bodies found in the SVZ of CCK1R^−/−^ and WT mice was similar (Figures 2C,F), suggesting that reduced proliferation in the SVZ leads principally to the generation of fewer cells of neural lineage.

### Fewer proliferating precursor cells in the SVZ and RMS of CCK1R^−/−^ female mice lead to a reduced number of mature neurons in the GL of the OB

**Figure 3:**
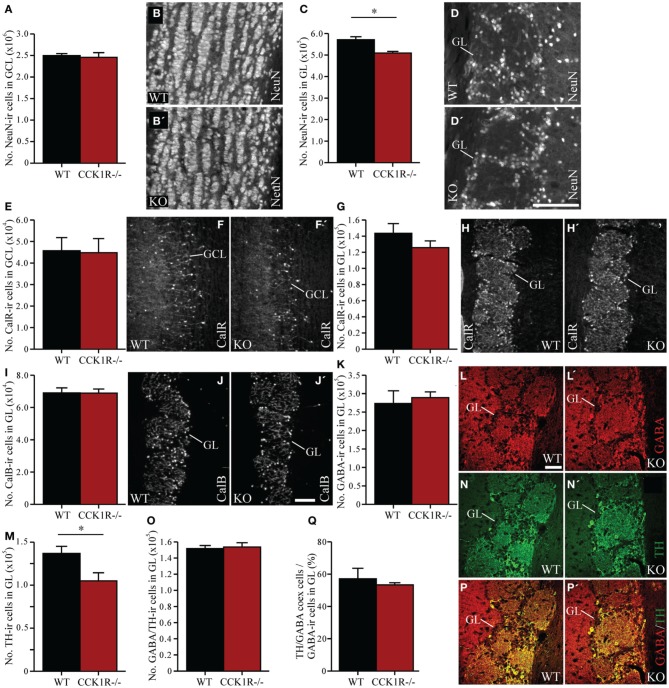
**The effect of reduced precursor proliferation in the SVZ of CCK1R^−/−^ female mice on subclasses of interneurons in the GCL and GL. (A,B)** Estimated number of NeuN-ir cell bodies in the GCL of wild-type and CCK1R^−/−^ mice **(A)**. Photomicrographs of NeuN-LI in the GCL of **(B)** wild-type and **(B′)** CCK1R^−/−^ mice. **(C,D)** Estimated number of NeuN-ir cell bodies in the GL of wild-type and CCK1R^−/−^ mice **(C)**. Photomicrographs of NeuN-LI in the GL of **(D)** wild-type and **(D′)** CCK1R^−/−^ mice. **(E,F)** Estimated number of calretinin-ir cell bodies in the GCL of wild-type and CCK1R^−/−^ mice **(E)**. Photomicrographs of calretinin-LI in the GCL of **(F)** wild-type and **(F′)** CCK1R^−/−^ mice. **(G,H)** Estimated number of calretinin-ir cell bodies in the GL of wild-type and CCK1R^−/−^ mice **(G)**. Photomicrographs of calretinin-LI in the GL of **(H)** wild-type and **(H′)** CCK1R^−/−^ mice. **(I,J)** Estimated number of calbindin-ir cell bodies in the GL of wild-type and CCK1R^−/−^ mice **(I)**. Photomicrographs of calbindin-LI in the GL of **(J)** wild-type and **(J′)** CCK1R^−/−^ mice. **(K,L)** Estimated number of GABA-ir cell bodies in the GL of wild-type and CCK1R^−/−^ mice **(K)**. Photomicrographs of GABA-LI in the GL of **(L)** wild-type and **(L′)** CCK1R^−/−^ mice. **(M,N)** Estimated number of TH-ir cell bodies in the GL of wild-type and CCK1R^−/−^ mice **(M)**. Photomicrographs of TH-LI in the GL of **(N)** wild-type and **(N′)** CCK1R^−/−^ mice. **(O)** Estimated number of GABA and TH double-labeled cell bodies in the GL of wild-type and CCK1R^−/−^ mice. **(P)** Double-immunofluorescence photomicrographs of TH-LI (green, **N**,**N′**) and GABA-LI (red, **L**, **L′**) in the GL of **(P)** wild-type and **(P′)** CCK1R^−/−^ mice. **(Q)** Estimated number of GABA-ir/TH-ir co-expressing cell bodies expressed as a proportion of the total number of GABA-ir cell bodies in the GL of wild-type and CCK1R^−/−^ mice. In all plots, black bars = wild-type female animals (*n* = 4), and red bars = CCK1R^−/−^ female mice (*n* = 4). CalB, calbindin; CalR, calretinin; GABA, γ-aminobutyric acid; GCL, granular cell layer of OB; GL, glomerular cell layer of OB; ir, immunoreactive; KO, CCK1R^−/−^; TH, tyrosine hydroxylase; WT, wild-type. Scale bars: **(D′)** = 100 μm, applies **(B,B′,D,D′)**; **(J′)** = 200 μm, applies **(F,F′,H,H′,J,J′)**; **(L)** = 50 μm, applies **(L,L′,N,N′,P,P′)**. ^*^corresponds to *P* < 0.05.

As cells born in the SVZ migrate along the RMS toward the OB, where they differentiate into local interneurons (Luskin, 1993; Lois and Alvarez-Buylla, 1994), the effect of reduced proliferation in the SVZ on the number of mature interneurons in the OB was examined. Mature OB interneurons were identified using an antibody against NeuN. Despite the reduction in the number of proliferating precursors in the SVZ of CCK1R^−/−^ mice, the number of NeuN-ir cell bodies in the GCL was similar to the number found in WT mice (Figures 3A,B). In the GL, however, the modest 11% decrease in NeuN-ir cell bodies in female CCK1R^−/−^ mice differed statistically from WT controls (Figures 3C,D).

### Subtypes of interneurons in the OB of female CCK1R^−/−^ mice remain unaffected, except for periglomerular TH-Ir interneurons

Subclasses of granular and periglomerular cells in the OB can be identified by expression of GABA, TH (Betarbet et al., 1996), calbindin, and calretinin (Rogers, 1992; Rogers and Resibois, 1992; De Marchis et al., 2007). We examined whether the reduction of cell proliferation in the SVZ of CCK1R^−/−^ mice affected the number of interneurons in each of these subclasses.

In the GCL of female CCK1R^−/−^ and WT mice, the number of calretinin-ir cell bodies was similar (Figures 3E,F). In the GL of female CCK1R^−/−^ mice, the number of calretinin-ir (Figures 3G,H), calbindin-ir (Figures 3I,J) and GABA-ir (Figures 3K,L) cell bodies was also similar to the number estimated in WT mice. However, the 23% reduction in the number of TH-ir cell bodies in the GL of CCK1R^−/−^ mice was statistically different from WT mice (Figures 3M,N). Double-labeling experiments revealed that the number of cells in the GL that co-expressed GABA- and TH-LI was similar in CCK 1R^−/−^ and WT mice (Figures 3O,P), as was the proportion of GABA-ir cells that co-expressed TH-LI (Figure 3Q). This suggests that the decreased number of TH-ir cell bodies in the GL of female CCK1R^−/−^ mice is not due to a reduced number of GABA/TH co-expressing cells, nor a decrease of TH expression in GABA-ir cells.

### No change in the number of newborn cells in the OB of CCK1R^−/−^ female mice

We next examined the effect of reduced SVZ precursor proliferation on the number of newborn cells that migrate to, integrate and survive in the GCL and GL of the OB. BrdU (50 mg/kg i.p.) was administered twice daily for 5 consecutive days. The mice were killed 42 days after the last BrdU administration, a suitable period for assessing the number of newly born cells that have matured and survived in the OB (Petreanu and Alvarez-Buylla, 2002; Winner et al., 2002; Lledo and Saghatelyan, 2005) (Figure 1B).

**Figure 4:**
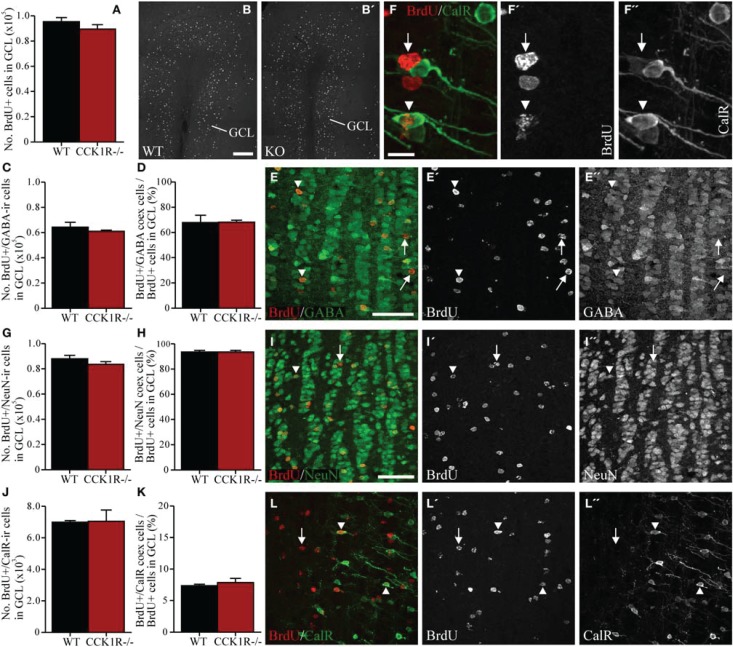
**Estimates of adult-born cells in the GCL of female CCK1R^−/−^ and wild-type mice.** BrdU (50 mg/kg i.p.) was administered twice daily for 5 consecutive days, and mice killed 42 days after the last BrdU administration (see *Protocol 2*, Figure 1B). **(A,B)** Estimated number of BrdU+ cell bodies in the GCL of wild-type and CCK1R^−/−^ mice **(A)**. Photomicrograph of BrdU+ cell bodies in the GCL of **(B)** wild-type and **(B′)** CCK1R^−/−^ mice. **(C–E)** Estimated number of BrdU+ and GABA-ir double-labeled cell bodies in the GCL of wild-type and CCK1R^−/−^ mice **(C)**. **(D)** Estimated number of BrdU+/GABA-ir co-expressing cell bodies expressed as a proportion of the total number of BrdU+ cell bodies in the GCL of wild-type and CCK1R^−/−^ mice. **(E)** Double-immunofluorescence confocal micrograph showing BrdU-LI (**E′**, red) and GABA-LI (**E″**, green) in the GCL of CCK1R^−/−^ mouse. **(G–I)** Estimated number of BrdU+ and NeuN-ir double-labeled cell bodies in the GCL of wild-type and CCK1R^−/−^ mice **(G)**. **(H)** Estimated number of BrdU+/NeuN-ir co-expressing cell bodies expressed as a proportion of the total number of BrdU+ cell bodies in the GCL of wild-type and CCK1R^−/−^ mice. **(I)** Double-immunofluorescence confocal micrograph showing BrdU-LI (**I′**, red) and NeuN-LI (**I″**, green) in the GCL of CCK1R^−/−^ mouse. **(J–L,F)** Estimated number of BrdU+ and calretinin-ir double-labeled cell bodies in the GCL of wild-type and CCK1R^−/−^ mice **(J)**. **(K)** Estimated number of BrdU+/calretinin-ir co-expressing cell bodies expressed as a proportion of the total number of BrdU+ cell bodies in the GCL of wild-type and CCK1R^−/−^ mice. **(L)** Double-immunofluorescence confocal micrograph showing BrdU-LI (**L′**, red) and calretinin-LI (**L″**, green) in the GCL of CCK1R^−/−^ mouse, shown at higher magnification in **(F), (F′)**, and **(F″)**, respectively. In all plots, black bars = female wild-type animals (*n* = 4), and red bars = female CCK1R^−/−^ mice (*n* = 4). Arrowheads point to double-labeled cell bodies, and arrows to single labeled BrdU+ cell bodies. BrdU, 5-bromo-2-deoxyuridine; CalR, calretinin; GABA, γ-aminobutyric acid; GCL, granular cell layer of OB; ir, immunoreactive; KO, CCK1R^−/−^; NeuN, neuronal nuclei; WT, wild-type; +, positive. Scale bars: **(B)** = 200 μm, applies **(B,B′)**; **(E)** = 50 μm, applies **(E,E′,E″)**; **(F)** = 10 μm, applies **(F,F′,F″)**; **(I)** = 50 μm, applies **(I,I′,I″,L,L′,L″)**.

In the GCL, 6% fewer BrdU+ cell bodies were observed in CCK1R^−/−^ female mice, however, this was not statistically different from WT (Figures 4A,B). Because interneurons in the GCL predominantly express GABA, double-immunofluorescence histochemistry for GABA and BrdU was performed (Figure 4E) to examine whether reduced SVZ proliferation in CCK1R^−/−^ mice led to a change in the number of BrdU+ newborn cells that had differentiated into GABA-ir interneurons. Forty-two days after the last BrdU administration, the number of cells in the GCL that were BrdU+ and contained GABA-LI was 5% lower in CCK1R^−/−^ mice, but not statistically different to WT mice (Figure 4C). The proportion of BrdU+ cell bodies in the GCL that co-expressed GABA-LI was also similar in WT (68%) and CCK1R^−/−^ mice (68%) (Figure 4D).

Double-immunofluorescence for BrdU and NeuN was also performed (Figure 4I). Forty-two days after the last BrdU administration, the number of cells in the GCL that were BrdU+ and contained NeuN-LI was statistically similar in CCK1R^−/−^ and WT mice (Figure 4G), as was the proportion of BrdU+ cell bodies in the GCL that co-expressed NeuN-LI (Figure 4H). Likewise, the number of cells in the GCL that were BrdU+ and contained calretinin-LI was similar in CCK1R^−/−^ and WT mice (Figures 4F,J,L), as was the proportion of BrdU+ cell bodies in the GCL that co-expressed calretinin-LI (Figure 4K).

**Figure 5:**
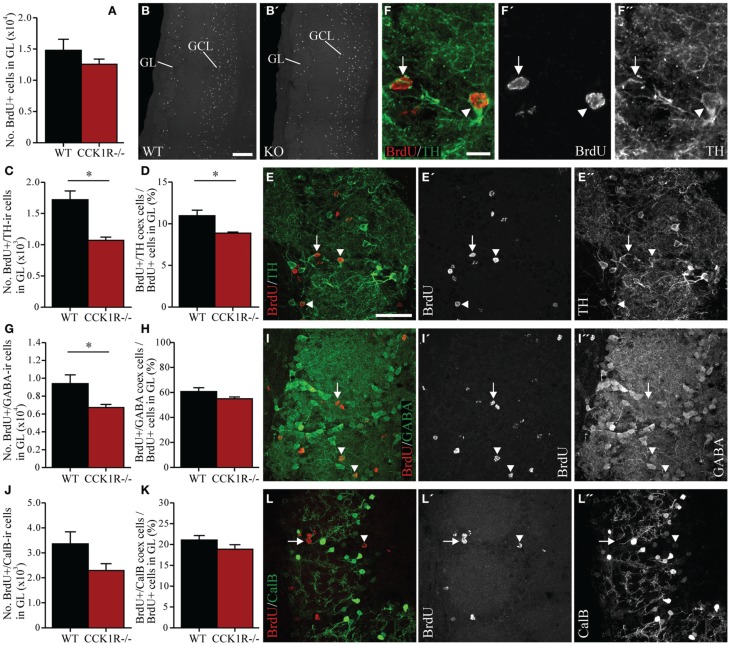
**Estimates of adult-born cells in the GL of female CCK1R^−/−^ and wild-type mice.** BrdU (50 mg/kg i.p.) was administered twice daily for 5 consecutive days, and mice killed 42 days after the last BrdU administration (see *Protocol 2*, Figure 1B). **(A,B)** Estimated number of BrdU+ cell bodies in the GL of wild-type and CCK1R^−/−^ mice **(A)**. Photomicrographs of BrdU+ cell bodies in the GL of **(B)** wild-type and **(B′)** CCK1R^−/−^ mice. **(C–F)** Estimated number of BrdU+ and TH-ir double-labeled cell bodies in the GL of wild-type and CCK1R^−/−^ mice **(C)**. **(D)** Estimated number of BrdU+/TH-ir co-expressing cell bodies expressed as a proportion of the total number of BrdU+ cell bodies in the GL of wild-type and CCK1R^−/−^ mice. **(E)** Double-immunofluorescence confocal micrograph of BrdU-LI (**E′**, red) and TH-LI (**E″**, green) in the GL of CCK1R^−/−^ mouse, shown at higher magnification in **(F)**, **(F′)**, and **(F″)**, respectively. **(G–I)** Estimated number of BrdU+ and GABA-ir double-labeled cell bodies in the GL of wild-type and CCK1R^−/−^ mice **(G)**. **(H)** Estimated number of BrdU+/GABA-ir co-expressing cell bodies expressed as a proportion of the total number of BrdU+ cell bodies in the GL of wild-type and CCK1R^−/−^ mice. **(I)** Double-immunofluorescence confocal micrograph showing BrdU-LI (**I′**, red) and GABA-LI (**I″**, green) in the GL of CCK1R^−/−^ mouse. **(J–L)** Estimated number of BrdU+ and calbindin-ir double-labeled cell bodies in the GL of wild-type and CCK1R^−/−^ mice **(J)**. **(K)** Estimated number of BrdU+/calbindin-ir co-expressing cell bodies expressed as a proportion of the total number of BrdU+ cell bodies in the GL of wild-type and CCK1R^−/−^ mice. **(L)** Double-immunofluorescence confocal micrograph showing BrdU-LI (**L′**, red) and calbindin-LI (**L″**, green) in the GL of CCK1R^−/−^ mouse. In all plots, black bars = female wild-type animals (*n* = 4), and red bars = female CCK1R^−/−^ mice (*n* = 4). Arrowheads point to double-labeled cell bodies, and arrows to single labeled BrdU+ cell bodies. BrdU, 5-bromo-2-deoxyuridine; CalB, calbindin; GABA, γ-aminobutyric acid; GCL, granular cell layer of OB; GL, glomerular cell layer of OB; ir, immunoreactive; KO, CCK1R^−/−^; TH, tyrosine hydroxylase; WT, wild-type; +, positive. Scale bars: **(B)** = 200 μm, applies **(B,B′)**; **(E)** = 50 μm, applies **(E,E′,E″,I,I′,I″,** and **L,L′,L″**); **(F)** = 10 μm, applies **(F,F′,F″)**. ^*^corresponds to *P* < 0.05.

In the GL, the number of BrdU+ cell bodies observed in female CCK1R^−/−^ mice was 15% lower, but not statistically different to the number observed in WT mice (Figures 5A,B). Because interneurons in the GL express TH, GABA and calbindin, double-immunofluorescence histochemistry for BrdU and these interneuronal subtypes was performed. Forty-two days after the last BrdU administration, the number of BrdU+ cells in the GL that co-labeled TH-LI in CCK1R^−/−^ mice was 38% lower than in WT mice (Figures 5C,E,F), and the proportion of BrdU+ cells co-expressing TH-LI reduced from 11% in WT mice, to 9% in CCK1R^−/−^ mice (Figure 5D). There were 29% fewer BrdU+ cells in the GL of CCK1R^−/−^ mice that co-labeled GABA-LI (Figures 5G,I), although the proportion of BrdU+ cell bodies that co-expressed GABA-LI remained similar to levels observed in WT mice (Figure 5H). No statistical change in the number BrdU+ cells in the GL that co-labeled calbindin-LI (Figures 5J,L), nor the proportion of BrdU+ cells that co-expressed calbindin-LI (Figure 5K) was observed when comparing WT and CCK1R^−/−^ mice. Finally, no BrdU+ cell bodies in GCL or GL of CCK1R^−/−^ or WT mice were found to co-express DCX-LI 42 days after the last BrdU administration (data not shown).

### Adult female CCK1R^−/−^ mice have a lower number of proliferating cells in the SGZ

**Figure 6:**
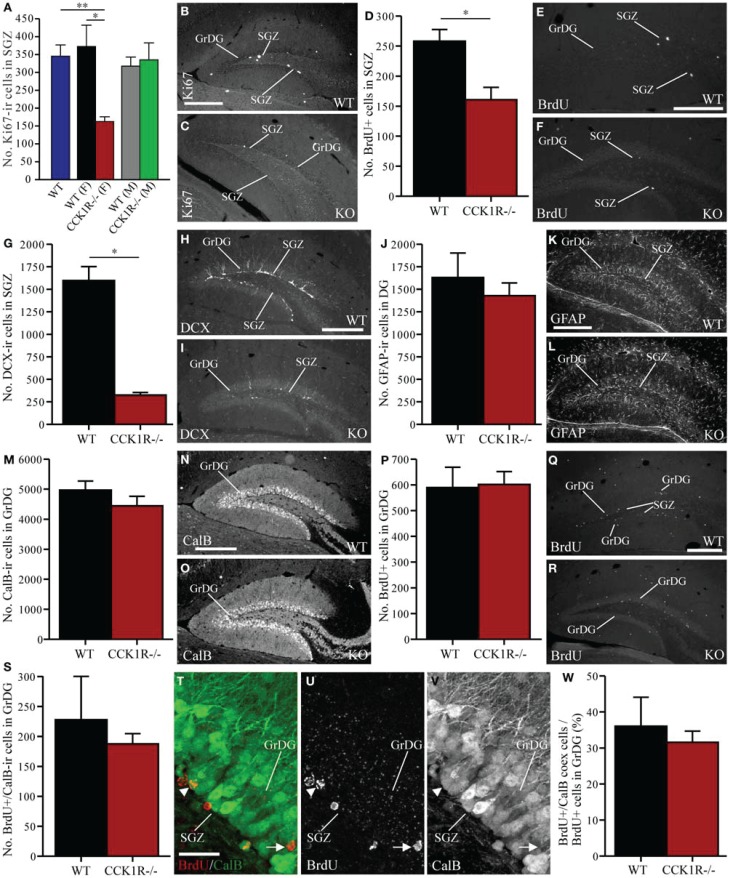
**Estimates of proliferating cells, neuroblasts, astrocytes, and mature neurons in the SGZ and GrDG of CCK1R^−/−^ and wild-type mice. (A–C)** Estimated number of Ki67-ir cell bodies in the SGZ of CCK1R^−/−^ and wild-type mice **(A)**. In **(A)**: WT blue bar = wild-type (male and female combined, *n* = 8); WT (F) black bar = female wild-type (*n* = 4); CCK1R^−/−^ (F) red bar = female CCK1R^−/−^ (*n* = 4); WT (M) grey bar = male wild-type (*n* = 4); and CCK1R^−/−^ (M) green bar = male CCK1R^−/−^ (*n* = 4). Photomicrograph of Ki67-ir cell bodies in the SGZ of **(B)** female wild-type and **(C)** female CCK1R^−/−^ mice. **(D–F)** Estimated number of BrdU+ cell bodies in the SGZ of female CCK1R^−/−^ and female wild-type mice **(D)** (see *Protocol 1*, Figure 1A). Photomicrograph of BrdU+ cell bodies in the SGZ of **(E)** female wild-type and **(F)** female CCK1R^−/−^ mice. **(G–I)** Estimated number of DCX-ir neuroblast cells in the SGZ of female CCK1R^−/−^ and female wild-type mice **(G)**. Photomicrograph of DCX-ir neuroblasts in the SGZ of **(H)** female wild-type and **(I)** female CCK1R^−/−^ mice. **(J–L)** Estimated number of GFAP-ir astrocytes in the DG of female CCK1R^−/−^ and female wild-type mice **(J)**. Photomicrograph of GFAP-ir astrocytes in the DG of **(K)** female wild-type and **(L)** female CCK1R^−/−^mice. **(M–O)** Estimated number of calbindin-ir cell bodies in the GrDG of female CCK1R^−/−^ and female wild-type mice **(M)**. Photomicrograph of calbindin-ir cell bodies in the GrDG of **(N)** female wild-type and **(O)** female CCK1R^−/−^ mice. **(P–R)** Estimated number of BrdU+ cell bodies in the GrDG of female wild-type and female CCK1R^−/−^ mice **(P)**. BrdU (50 mg/kg i.p.) was administered twice daily for 5 consecutive days, and mice killed 42 days after the last BrdU administration (see *Protocol 2*, Figure 1B). Photomicrograph of BrdU+ cell bodies in the GrDG of **(Q)** female wild-type and **(R)** female CCK1R^−/−^ mice. **(S–W)** Estimated number of BrdU+ and calbindin-ir double-labeled cell bodies in the GrDG of female wild-type and female CCK1R^−/−^ mice **(S)**. **(T)** Double-immunofluorescence confocal micrograph showing BrdU-LI (**U**, red) and calbindin-LI (**V**, green) in the GrDG of female wild-type mouse. Arrowheads point to BrdU/calbindin double-labeled cell bodies. Arrows point to single-labeled BrdU+ cell bodies. **(W)** Estimated number of BrdU+/calbindin-ir co-expressing cell bodies expressed as a proportion of the total number of BrdU+ cell bodies in the GrDG of female wild-type and female CCK1R^−/−^ mice. In all plots, black bars = female wild-type animals (*n* = 4), and red bars = female CCK1R^−/−^ mice (*n* = 4). BrdU, 5-bromo-2-deoxyuridine; CalB, calbindin; DCX, doublecortin; GFAP, glial fibrillary acidic protein; GrDG, granule cell layer of DG; ir, immunoreactive; KO, CCK1R^−/−^; SGZ, subgranular zone of DG; WT, wild-type; +, positive. Scale bars: **(B)** = 250 μm, applies **(B,C)**; **(E)** = 200 μm, applies **(E,F)**; **(H)** = 250 μm, applies **(H,I)**; **(K)** = 250 μm, applies **(K,L)**; **(N)** = 250 μm, applies **(N,O)**; **(Q)** = 200 μm, applies **(Q,R)**; **(T)** = 25 μm, applies **(T–V)**. ^*^corresponds to *P* < 0.05 (student *t*-test); ^**^corresponds to *P* < 0.05 (ANOVA with Tukey multiple comparisons test).

To examine whether CCK1R deletion leads to a change in the number of proliferating cells in the DG, immunoreactivity against Ki67 was performed to identify proliferating cells in the SGZ (i.e., cells bordering the polymorph and granule layers of the DG), who subsequently differentiate into granule cells in the DG (Eriksson et al., 1998; Cameron and McKay, 2001; Kee et al., 2002; Ming and Song, 2011; von Bohlen Und Halbach, 2011). Ki67-ir cell bodies in the SGZ of female CCK1R^−/−^ mice were down 56% in comparison to female WT controls (Figures 6A–C). A statistical difference in the number of Ki67 cells in the SGZ was observed when comparing WT and female CCK1R^−/−^ mice and female CCK1R^−/−^ vs. male CCK1R^−/−^ mice, while WT and male CCK1R^−/−^ mice were statistically similar, as were male and female WT mice (Figure 6A, ANOVA with Tukey multiple comparisons test).

BrdU (150 mg/kg i.p.) was also injected into mice 2 h prior to their death (Figure 1A). BrdU+ cell bodies were found in the SGZ, where their number in female CCK1R^−/−^ mice was 38% lower than in female WT mice (Figures 6D–F).

### Lower numbers of DCX-ir neuroblasts in the dentate gyrus of female CCK1R^−/−^ mice

Because neural progenitors in the SGZ give rise to immature neurons (Van Praag et al., 2002; Ming and Song, 2005), we examined whether the number of DCX-ir neuroblasts in the DG were altered in female CCK1R^−/−^ mice. DCX-ir cell bodies were observed within the SGZ and GrDG, with dendritic processes extending through the GrDG, and into the molecular layer (Figure 6H). There were 80% fewer DCX-ir neuroblasts in the SGZ and GrDG of CCK1R^−/−^ mice than in WT mice (Figures 6G–I).

### Similar numbers of GFAP-ir astrocytes in the dentate gyrus of female CCK1R^−/−^ mice

Immunohistochemistry for GFAP was performed to determine whether reduced proliferation in the SGZ also influenced the number of astrocytes in the DG. Strong GFAP-LI was observed in the DG of CCK1R^−/−^ and WT mice (Figures 6K,L), and the number of GFAP-ir astrocytes in the SGZ and GrDG was quantified. We found 12% fewer GFAP-ir astrocytes in CCK1R^−/−^ mice which was not statistically different from WT controls (Figure 6J), suggesting that reduced proliferation in the SGZ of CCK1R^−/−^ female mice leads principally to the generation of fewer cells of neural lineage, without affecting the generation of astrocytes.

### Similar numbers of calbindin-Ir cell bodies in the dentate gyrus of female CCK1R^−/−^ mice

Newly generated neurons in the SGZ migrate a short distance to the GrDG (Ming and Song, 2005), where they mature into neurons that express calbindin (Sloviter, 1989; Markakis and Gage, 1999; Van Praag et al., 2002). Immunohistochemistry for calbindin was performed to determine whether reduced proliferation in the SGZ also led to fewer calbindin-ir neurons in the GrDG. Although the number of calbindin-ir cell bodies in CCK1R^−/−^ mice was 10% fewer than in WT mice (Figures 6M–O), this change was not statistically different.

### No change in the number of mature newborn cells in the dentate gyrus of CCK1R^−/−^ female mice

We next examined the effect of reduced SGZ precursor proliferation on the number of newborn cells that integrate and survive in the GrDG. BrdU (50 mg/kg i.p.) was administered twice daily for 5 consecutive days, and the mice were killed 42 days after the last BrdU administration (Figure 1B), a suitable period for assessing the number of newly born cells that have survived and matured in the GrDG (Ming and Song, 2005).

In the GrDG, the number of BrdU+ cell bodies observed in CCK1R^−/−^ female mice was similar to the number observed in WT mice (Figures 6P–R). Because the majority of mature neurons in the GrDG express calbindin-LI, double-immunofluorescence histochemistry for calbindin and BrdU was performed to examine whether reduced SGZ proliferation in CCK1R^−/−^ mice led to a change in the number of BrdU+ newborn cells that had differentiated into calbindin-ir interneurons. Forty-two days after the last BrdU administration, the number of cells in the GrDG that were BrdU+ and contained calbindin-LI was 18% fewer in CCK1R^−/−^ mice, but not statistically different to WT mice (Figures 6S–V). A 12% decrease in the proportion of BrdU+ cell bodies in the GCL that co-expressed calbindin-LI was found in CCK1R^−/−^ mice (Figure 6W), which also was not statistically different to the proportion found in WT mice. Finally, 42 days after the last BrdU administration, no BrdU+ cell bodies in the GrDG of CCK1R^−/−^ or WT mice were found to co-express DCX-LI (data not shown).

## Discussion

Using genetically modified mice, we provide evidence that CCK, by actions mediated through the CCK1R, can regulate cell proliferation in the adult mouse SVZ and SGZ. Female mice lacking these receptors were found to have fewer proliferating cells and less migratory neuroblasts in the SVZ, RMS and SGZ. Our data indicate that the reduced number of proliferating precursors in the SVZ and SGZ of CCK1R^−/−^ female mice had a discrete effect on the number of mature neurons in the OB: the number NeuN-ir cell bodies and TH-ir interneurons in the GL of the OB was reduced, as was the number of BrdU+ cell bodies in the GL that co-expressed TH-LI or GABA-LI.

In general, our results point to a regulation of neurogenesis in the adult brain, so that a steady neuronal population is maintained in the OB and GrDG, irrespective of the number of proliferating cells in the SVZ or SGZ, or their rate of proliferation. Despite the reduction in proliferating cells and neuroblasts in the SVZ, RMS and SGZ of female CCK1R^−/−^ mice, the number of adult-born BrdU+ cell bodies in the GL, GCL and GrDG was similar to WT mice 42 days after the last BrdU pulse. This coincided with a similar number of adult-born BrdU+ neurons in CCK1R^−/−^ and WT mice that expressed: NeuN-, GABA or calretinin-LI in the GCL; calbindin-LI in the GCL; and calbindin-LI in the GrDG. The capacity for maintaining the number of adult-born cells that survived and integrated into the circuitry of the OB and GrDG, despite the lower availability of adult-born cells, led to the number of mature NeuN-ir and calretinin-ir cell bodies in the GCL, calbindin-ir, calretinin-ir and GABA-ir cell bodies in the GL, and calbindin-ir cell bodies in the GrDG remaining the same in CCK1R^−/−^ and WT mice.

Our findings are in contrast with previous reports that used agents that cause permanent and often complete suppression of proliferation in the SVZ or SGZ, and that examined the effects of reduced SVZ/SGZ proliferation over a longer period. In previous work, genetic ablation of newly formed neurons in adult mice led to a progressive reduction in the number of DCX-ir neuroblasts in the SVZ and a gradual decrease in OB granule cells 3–12 weeks after ablation (Imayoshi et al., 2008). Similarly, x-ray irradiation that reduced adult-born cells in the SVZ by 96% led to a 20% decrease in OB granule cells 8 weeks after irradiation (Valley et al., 2009). Here, we report a ~40% reduction in the number of proliferating cells and 29% fewer DCX-ir neuroblasts in the SVZ/RMS of female CCK1R^−/−^ mice, but no consequent change in the number of OB granule cells.

A key difference in models used previously is the almost complete suppression of proliferating cells and neuroblasts in the SVZ/RMS that was induced in adult animals (Imayoshi et al., 2008; Valley et al., 2009). This compares to the permanent, but less vigorous, reduction of proliferating cells and neuroblasts that arises from the developmental deletion of the CCK1R, which allows for the generation of a lower than normal number of adult-born cells that still have the capacity to migrate to and integrate into OB circuitry. Thus, an explanation for our observation of no change in OB granule cells when SVZ proliferation is reduced, is a greater rate of survival of adult-born cells generated in the SVZ and RMS of CCK1R^−/−^ mice. In support of this notion, the number of mature BrdU+ interneurons in the GCL that expressed GABA-LI, calretinin-LI or NeuN-LI was similar in CCK1R^−/−^ and WT mice 42 days after the last BrdU pulse.

Under normal conditions, adult-born cells generated in the SGZ add to the number of neurons in the GrDG over time (Bayer et al., 1982; Dayer et al., 2003; Imayoshi et al., 2008), whereas ablation of neurogenesis prevents such an increase so that the number and density of neurons in the GrDG remain constant (Imayoshi et al., 2008). If the same principles were to apply here, we would expect the number of neurons in the GrDG of CCK1R^−/−^ mice to be lower than the numbers in WT mice. However, we found a similar number of calbindin-ir cell bodies in the GrDG of CCK1R^−/−^ and WT mice, despite the reduction in proliferating cells (38 and 56% fewer BrdU+ and Ki67-ir cell bodies, respectively) and DCX-ir neuroblasts (80%) in the SGZ. Again, this may be attributable to the increased rate of survival of adult-born cells generated in the SGZ, where 42 days after the last injection of BrdU, a similar number of BrdU+ cell bodies and BrdU cell bodies that expressed calbindin-ir was observed in the GrDG of CCK1R^−/−^ and WT mice.

The situation was different in the GL of the OB, where fewer TH-ir interneurons and BrdU+ cell bodies that expressed either TH-LI or GABA-LI were found in CCK1R^−/−^ mice. No change in the number of calbindin-ir cell bodies, or BrdU+/calbindin-ir interneurons was observed in the GL however, suggesting that CCK1R deletion and/or reduced SVZ proliferation has a limited influence on calbindin-expressing interneurons, most of which are generated early in life (De Marchis et al., 2007). In contrast, TH-ir and calretinin-ir cell bodies in the GL are predominantly generated in the adult (McLean and Shipley, 1988; Winner et al., 2002; De Marchis et al., 2007) and the number of TH-ir cell bodies was affected in adult female CCK1R^−/−^ mice. [N.B. the non-statistical trend for a reduction in calretinin-ir cell bodies in the GL of CCK1R^−/−^ mice, and previous qualitative results showing reduced numbers of calretinin-ir cell bodies in CCK1R^−/−^ mice (Stanić et al., 2008).] Recently, we also found changes to the numbers of calretinin-ir and TH-ir, but not calbindin-ir, cell bodies in the GL of adult mice following an induced reduction of proliferation in the SVZ (Sui et al., 2012), and odor deprivation reduces TH expression in the GL, without affecting the GABA, calbindin and calretinin phenotypes (Stone et al., 1991; Baker et al., 1993; Bastien-Dionne et al., 2010). Our results therefore suggest that the CCK1 receptor may play an important role in modulating the generation and/or survival of TH (and calretinin) interneurons in the GL of the OB.

While plasticity in the population of TH-ir interneurons may reflect their ability to adapt to continuously changing odor environments (Doetsch and Hen, 2005), changes in subtypes of interneurons present in the OB may alter the complex circuitry that exists within the OB. This includes the intricate arrangement of dendrites in the external plexiform layer that are derived from mitral, granule and tufted cells that engage in dendro-dendritic reciprocal synaptic interactions with each other (Rall et al., 1966; Shepherd, 1972; Jackowski et al., 1978; Shipley et al., 1996; Stanić et al., 2010), and the interactions of periglomerular cells in the glomerular layer (Shipley and Ennis, 1996; Kosaka and Kosaka, 2005). Because less TH-ir interneurons were integrated into OB circuitry, functional properties of mitral and tufted cells [e.g., their odorant-evoked firing properties (Nagayama et al., 2004)] and the timing of the transmission of olfactory information and bulbar output may be altered. Thus, it would be interesting to determine whether deficits in olfactory functioning, e.g., short- and long-term odor memory, odor discrimination and fear conditioning (Gheusi et al., 2000; Rochefort et al., 2002; Lazarini et al., 2009; Valley et al., 2009), exist in adult CCK1R^−/−^ female mice.

Proliferating cells and neuroblasts were reduced only in female CCK1R^−/−^ mice. It is possible that this sex difference is related to estrous cycle influences (Ormerod and Galea, 2001) because, for example, estrus induction is associated with increased numbers of dividing cells in the SVZ/RMS of prairie voles (Smith et al., 2001), and adult female rats produce more cells during proestrus, compared with estrus and diestrus (Tanapat et al., 1999). Furthermore, levels of CCK fluctuate in the brain during a normal estrous cycle (Hilke et al., 2007). Thus, lower levels of CCK during pro-estrus, combined with absence of the CCK1R, may reduce the rate of cell division in neurogenic regions of the female mouse brain. However, we do not favor this explanation because, in C57BL6 mice, proliferation or neurogenesis in the SGZ is not influenced by the estrous cycle or after ovariectomy (Lagace et al., 2007), and no gender differences in hippocampal proliferation or neurogenesis was observed here, or previously in mice (Lagace et al., 2007; Manning et al., 2012). Thus, it is unlikely that the estrous cycle and fluctuating estradiol levels contributed to the lower numbers of proliferating cells and neuroblasts observed in female CCK1R^−/−^ mice. The neurochemical mechanisms underlying reduced proliferating cells and neuroblasts in female CCK1R^−/−^ mice remains unclear and awaits future investigations.

In conclusion, we show a reduction of proliferation in the SVZ and SGZ of adult female CCK1R^−/−^ mice that does not alter the number of mature neurons in the OB and GrDG, except for TH-ir interneurons in the GL of the OB. Despite these mice having fewer proliferating cells and neuroblasts, we proposed that the numbers of mature neurons are maintained in the OB and GrDG due to the enhanced survival of neuroblasts and mature neurons that integrate into the circuitry of the OB and DG. Further investigations are needed to understand the role of these “longer-surviving” adult-born cells on the circuitry and function of the OB and DG.

### Conflict of interest statement

The authors declare that the research was conducted in the absence of any commercial or financial relationships that could be construed as a potential conflict of interest.
